# Effects of lower back foam rolling on the pressure pain threshold and the range of motion of the lumbar spine in healthy individuals

**DOI:** 10.3389/fphys.2024.1476342

**Published:** 2024-09-25

**Authors:** Julia Fijavž, Maja Frangež, Renata Vauhnik

**Affiliations:** ^1^ Faculty of Health Sciences, University of Ljubljana, Ljubljana, Slovenia; ^2^ Institute of Medical Rehabilitation, University Medical Centre Ljubljana, Ljubljana, Slovenia

**Keywords:** fascia, soft-tissue release, superficial back line, foam roller, pain, flexibility, lumbar spine

## Abstract

**Introduction:**

The aim of this study was to determine the short- and long-term effects of foam rolling (FR) on the pressure pain threshold and the range of motion of the lumbar spine in healthy subjects.

**Methods:**

43 healthy subjects without back problems were randomly assigned to an experimental group (EG) or a control group (CG). The subjects in the EG underwent a 4-week FR program (12 sessions). The subjects in the CG received no intervention. Range of motion was measured using the modified-modified Schober test for flexion and fingertip-to-floor distance for lateral flexion. The pressure pain threshold was measured with a hand-held pressure algometer. The measurements were taken before and after the first FR, after the 4-week program and at the 1-, 3- and 6-month follow-up. The significance level was set at p ≤ 0.05 and the desired power of the test was 92%.

**Results:**

We found an improvement in flexion (p = 0.03) and lateral flexion (p < 0.001) in the EG after the first FR and recorded a significant improvement in all measured variables (flexion, lateral flexion and algometry: p < 0.001) at the end of the entire 4-week program. The effects were noticeable up to 6 months after the end of the program (p ≤ 0.03) and were statistically significantly better than in the CG (p ≤ 0.04). The calculated Cohen's d value was 1.15 for flexion, 1.06 for lateral flexion and 0.98 for algometry, which represents a large effect size.

**Discussion:**

FR improves the pressure pain threshold and mobility of the lumbar spine in healthy subjects. The effects are noticeable at least 6 months after the end of an FR program.

## 1 Introduction

Fascia is a connective tissue that surrounds and connects all the muscles, bones and organs in our body (Findley et al., 2012). Fascia is very strong, but also plastic (Schleip, 2003; Findley et al., 2012). It is thought that changes in the arrangement of the fascia can be caused by inactivity, overload, injury, inflammation or disease (Stecco et al., 2011). As fascia can transmit tension and also have a proprioceptive and nociceptive function, disorders in one part of the body could radiate to distant anatomical structures via myofascial meridians and cause tension or pain there (Myers, 2014; Wilke et al., 2016).

Many manual therapy techniques focus on the fascia. It is claimed that by applying manual pressure, changes in fascial density, tone, viscosity or alignment occur, which consequently reduce pain and improve mobility (Schleip, 2003; Beardsley and Škarabot, 2015). Previous research has shown that people with chronic low back pain have reduced mobility of the thoracolumbar fascia and consequently reduced mobility of the lumbar spine (Langevin et al., 2011). Furthermore, research suggests that there is a link between increased muscle stiffness, decreased trunk flexibility and the risk of low back pain (Ito et al., 2023; Vatovec and Voglar, 2024). It is believed that the above problems can be addressed through foam rolling (FR). This technique has recently become very popular and affordable, allowing individuals to perform their own therapeutic sessions designed to mimic the effects of manual therapy (Beardsley and Škarabot, 2015; Krause et al., 2019). The most commonly used tool for FR is a foam roller. Although the optimal hardness of FR tools has not been well researched, existing studies and the authors’ clinical experience favor the use of softer tools (Miller, 2020).

Previous research has found many positive effects of FR, including: improved hip, knee and ankle mobility (Wilke et al., 2019; Hendricks et al., 2020; Skinner et al., 2020; Fauris et al., 2021; Kasahara et al., 2022; Kasahara et al., 2024); increase in lower limb pressure pain threshold (Wiewelhove et al., 2019; Hendricks et al., 2020; Kasahara et al., 2022; Kasahara et al., 2024), reduction in pain in people with fibromyalgia (Ceca et al., 2017); reduction of muscle fatigue or delayed onset muscle soreness (DOMS) and accelerated recovery after exercise (Hendricks et al., 2020; Skinner et al., 2020; Michalak et al., 2024); improved coordination of movement (David et al., 2019); reduced arterial stiffness, improved endothelial function and blood flow (Okamoto et al., 2014; Hotfiel et al., 2017; Alonso-Calvete et al., 2021); increased parasympathetic nervous system activity (Beardsley and Škarabot, 2015; Lastova et al., 2018) etc. Research also shows that FR does not impair athletic performance (Beardsley and Škarabot, 2015; Pagaduan et al., 2022). This is particularly beneficial for athletes who want a short-term improvement in flexibility without the loss of performance associated with static stretching (Beardsley and Škarabot, 2015). The advantage of FR is also that it generally has no effect on the deterioration of muscle strength, jump height and sprint time (Behm and Wilke, 2019). Peacock et al. (2014) even find that FR acutely improves muscle strength, explosiveness, agility and speed.

The aim of this study is to determine the short- and long-term effects of FR with a foam roller on the pressure pain threshold and range of motion of the lumbar spine in healthy subjects. We hypothesized that FR performed with a foam roller increases the range of motion in the direction of flexion and lateral flexion and increases the pressure pain threshold of the lumbar spine; that the effects are already noticeable after the first FR and that they still persist at 1, 3 and 6 months follow up.

## 2 Materials and methods

### 2.1 Experimental approach

**FIGURE 1:**
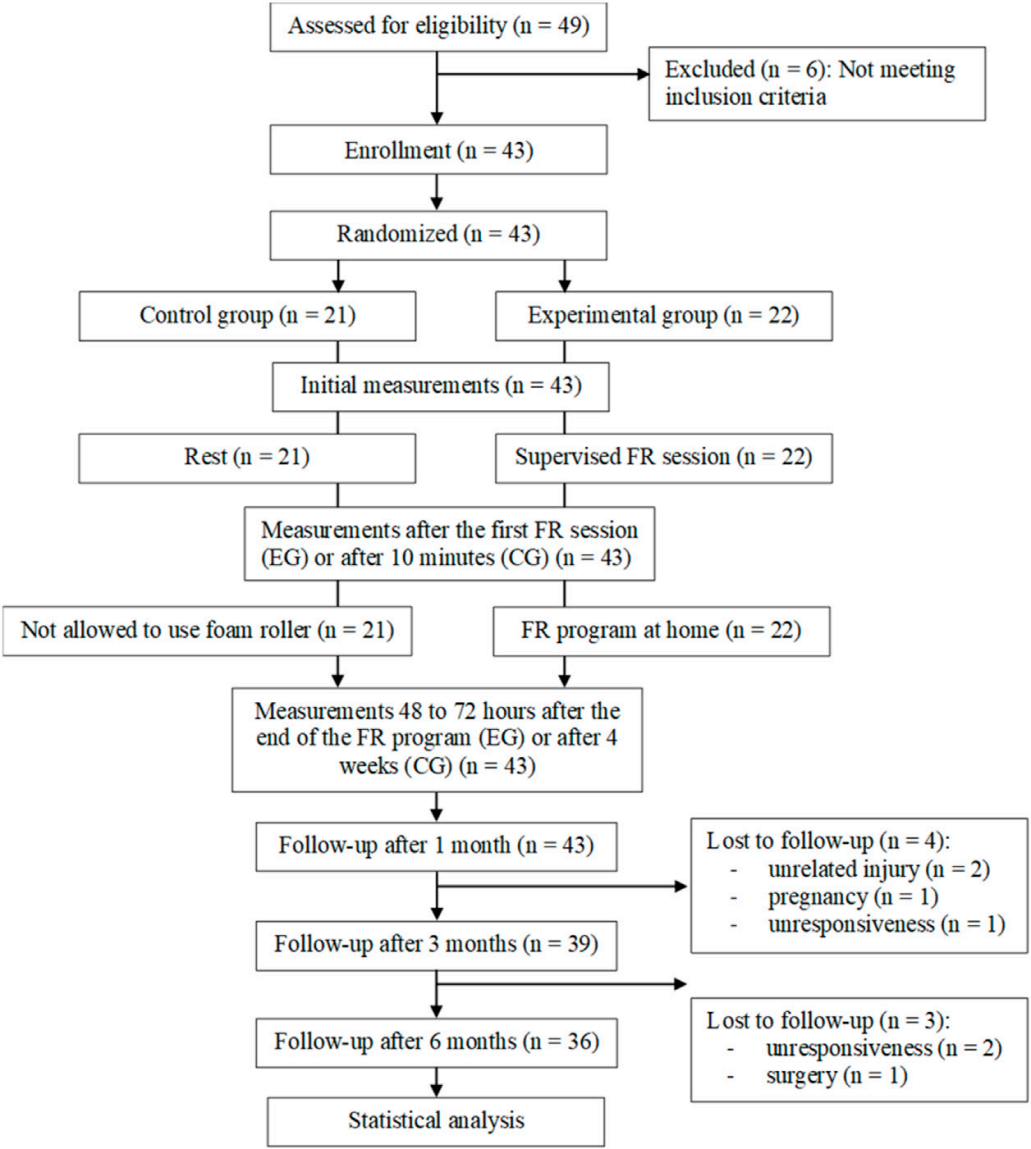
CONSORT diagram with participant flow.

This study is a randomized controlled trial that investigated the short-and long-term effects of FR on the pressure pain threshold and flexibility of the lumbar spine in healthy subjects. The study design is illustrated in Figure 1. It was conducted at the local community premises in Slovenske Konjice and lasted 9 months. Subjects who met the inclusion criteria were asked to complete a questionnaire to obtain their demographic data (gender, age, body mass, height). They were then randomly assigned to the experimental group (EG) or the control group (CG) using a randomization app (RandomIZE–Randomization Tool).

The EG performed FR 3 times per week, for 4 weeks, i.e., a total of 12 sessions. The subjects followed the program in full and completed 100% of the sessions. Tests were performed before and after the first FR (to determine the short-term effects), 48–72 h after the end of the 12 sessions, i.e., after 4 weeks, and at follow-up after 1, 3 and 6 months (to determine the long-term effects).

The CG did not undergo any intervention and was not allowed to use the foam roller until data collection was completed. The tests were conducted at the same intervals as in the, EG. The first two testing, which the EG subjects underwent before and after the first FR, were carried out 10 minutes apart in the CG. The time corresponded to the duration of the first FR. During this time, the subjects in the CG were asked to rest.

We measured the range of motion of the lumbar spine with the modified-modified Schober test for flexion and fingertip-to-floor distance for lateral flexion. The pressure pain threshold was measured with a hand-held pressure algometer.

### 2.2 Subjects

43 healthy subjects over the age of 18 (14 men, 29 women; age: 31.6 ± 9.8 years, age range: 18–60 years, height: 172.7 ± 9.2 cm, weight: 72.3 ± 14.6 kg, body mass index: 24.1 ± 3.6 kg/m^2^, all expressed as mean ± SD) volunteered to participate in the study after a public invitation was posted on social networks. Exclusion criteria were previous surgery or spine injury, the presence of acute or chronic back pain, the presence of musculoskeletal, neurological, systemic or metabolic diseases that could affect the range of motion or the pressure pain threshold measurements; the use of painkillers and anti-inflammatory drugs or injections; pregnancy. Prior to testing, all participants were fully informed about the experimental procedures and the purpose of the study and all have given written informed consent before participation. Participants were randomly assigned to either EG (n = 22) or CG (n = 21). At baseline, there were no statistically significant differences between the groups. The demographic characteristics of the included subjects are presented in Table 1. This study was approved by the National Medical Ethics Committee of the Republic of Slovenia (Ethical approval No: 0120-405/2021/3).

**TABLE 1:** Demographic characteristics of the included subjects.

	Gender m (f)	Age (years) x ± SD (min–max)	Height (cm) x ± SD (min–max)	Weight (kg) x ± SD (min–max)	BMI x ± SD (min–max)
Control group (n = 21)	5 (16)	33.5 ± 10.6 (22–60)	171.4 ± 8.5 (161–195)	72.7 ± 14.1 (54–104)	24.7 ± 3.9 (20–34)
Experimental group (n = 22)	9 (13)	29.7 ± 8.8 (18–52)	173.9 ± 9.8 (162–193)	72.0 ± 15.5 (51–105)	23.6 ± 3.2 (19–32)
p	0.38	0.19	0.39	0.72	0.46

Legend: m, male; f, female; x = mean; SD, standard deviation; n, number of participants; BMI, body mass index; *p* = level of significance.

### 2.3 Procedures

#### 2.3.1 Training program

The subjects in the EG performed the first FR session under the supervision of the researcher. They were then given printed instructions with a calendar to monitor regular performance of the exercises and a foam roller to use at home, where they continued with the FR program three times a week (counting the first supervised session), for 4 weeks, i.e. 12 sessions in total. They had at least one rest day between FR days. Our protocol is consistent with the findings of Pagaduan et al. (2022) where it was pointed out that it is necessary to perform foam rolling at a frequency of 3 times per week for long-term improvement in mobility and of Konrad et al. (2022) that FR should be performed for at least 4 weeks. Subjects were using Blackroll^®^ Med, a softer version of the foam roller, as recommended in the literature (Miller, 2020). The printed instructions were standardized for all subjects in the EG. They were instructed to perform the exercises in the evening and with such pressure on the foam roller that they caused no or minimal pain, i.e., up to a score of 3/10 on a numeric rating scale. The speed of foam-rolling was also standardized using a metronome set to a frequency of 60 (1 stroke per second). The exercises targeted the superficial back myofascial line according to Myers (2014). Subjects performed five FR exercises on the following areas: plantar fascia, posterior calf area, posterior thigh area, buttocks and lower back (see Supplementary Material which demonstrates the exercises used).

#### 2.3.2 Testing methods

The flexion ROM of the lumbar spine was measured using the modified-modified Schober test (van Adrichem and van der Korst, 1973; Malik et al., 2016). The subject stood in a neutral position with feet shoulder-width apart. We marked two points on the lumbar spine - the first point was at the point between the two SIPS and the second point was marked 15 cm higher, following the spinal curve. For the flexion test, the subject bent forwards towards the floor with knees straight and the distance between the points was measured again. The result was the difference between the final value and the initial value. Amjad et al. (2022) found an excellent reliability (ICC = 0.91–0.93) of the test for assessing lumbar spine flexion in healthy subjects. Tousignant et al. (2005) reported moderate validity (r = 0.67) of the test in patients with low back pain.

Lateral flexion range of motion was measured with a measuring tape (Mellin, 1986). The subjects stood with their backs to the wall and legs together. Their palms were placed on the lateral side of the thighs with the fingers extended. In this position, we measured the distance between the third finger and the floor. The subjects then performed a movement in the direction of lateral flexion and tried to get as close as possible to the ground with their fingers, after which we measured the distance between the third finger and the ground again. While performing the movement, the subjects had to maintain contact with the wall, were not allowed to rotate the torso, had to have their knees straight and feet on the ground at all times. The range of motion was represented by the difference between the start and end values. As a result we used the average values for the left and right sides. This is a widely used method that measures mobility of the entire thoracolumbar spine (Ng et al., 2001) and has a high test-retest reliability (ICC = 0.90–0.94) (Flavell et al., 2016).

The pressure pain threshold was measured using a manual pressure algometer (Baseline, Italy), with a 1 cm^2^ rubber attachment. The subject was in the prone position. We tested four points on the lumbar spine, placed symmetrically 2 cm to the left and right of the spinous processes of the vertebrae. When the pressure became uncomfortable for the subject, the measurement was stopped and the value was obtained. As recommended by literature (Aboodarda et al., 2015), we performed the measurements twice with a 1-min break in between and used the average of these two measurements as the result. Middlebrook et al. (2020) found high inter-rater reliability (ICC = 0.76–0.87) and excellent intra-rater reliability (ICC = 0.90–0 .93; SEM = 5.2–6.9 N) when performing the test at the spine area.

### 2.4 Statistical analysis

Statistical analysis was performed with RStudio (RStudio Inc., v2022.07.1, Boston, MA, ZDA; www.rstudio.com). The Shapiro-Wilk test was used to test the normality of the distribution of demographic data. The data were presented as mean and standard deviation. The groups were randomized at baseline which ensured comparability with respect to all covariates. To demonstrate the absence of differences between groups at baseline, we used the χ2 test for gender and the Mann-Whitney test for age, height, body mass, and BMI. Linear mixed models for repeated measures were used to determine differences between group means and between means within groups at different time points. For the results of the flexion, lateral flexion and algometry measurements, we first checked the assumptions of the linear mixed models and identified possible non-normality using the residuals of the models and the Q-Q plots. Deviations from normality were found in the algometry measurements, so that a logarithmic transformation of the data was required before further statistical analysis (checked with a Box-Cox transformation). The statistical significance level for all analyses was set at p ≤ 0.05. The Cohen’s coefficient d was used to calculate the effect size. Values up to 0.2 stand for a small effect size, up to 0.5 for a moderate effect size and over 0.8 for a large effect size (Cohen, 1988). We used simulations to perform a power analysis for the entire study to determine the probability of a type II error. The desired power of the test should be above 0.80 or 80% (Stare, 2007), in our case it was 92%.

## 3 Results

There were no statistically significant differences between the groups in the measurement of flexion at the beginning of the study (p = 0.21). Within the EG, we found a statistically significant improvement in flexion after the first FR session (p = 0.03). The improvement was greatest at the end of the entire 4-week FR program (average 1.5 cm; p < 0.001) and was also noticeable at all follow-up measurements, including the last at 6 months (p = 0.02). There were no statistically significant differences within the CG when the results were compared with the initial measurements (p > 0.02). Comparison of the results between the groups showed that flexion in the, EG improved statistically significantly at the end of the 4-week program (mean 1.1 cm compared to the CG; p < 0.001). The difference between the groups was still noticeable up to 6 months after the end of the FR program (p < 0.01).

In the measurements of lateral flexion, there were statistically significant differences between the groups at the beginning, namely that the EG initially achieved worse results compared to the CG (p < 0.001). In the EG, we found a statistically significant improvement in flexion after the first FR session (p < 0.001). The improvement was greatest at the end of the entire 4-week FR program (mean 7.3 cm; p < 0.001) and was also noticeable at all follow-up measurements, including the last one after 6 months (p < 0.01). There was no statistically significant improvement in lateral flexion within the CG when results were compared to baseline measurements, but there was a statistically significant worsening of measurement results at the 3- and 6-month follow-up (p < 0.01). Comparison of the results between the groups showed that lateral flexion in the EG improved statistically significantly at the end of the 4-week program (average 5.8 cm compared to the CG; p < 0.001). A statistically significant difference between the groups was also observed 3 and 6 months after the end of the FR program (p < 0.01).

The results of short-term effects are presented in Table 2, of effects at the end of the 4-week FR program in Table 3 and of long-term effects in Table 4.

**TABLE 2:** Short-term effects – comparison of results after vs. before the first FR.

	Flexion (cm)	Lateral flexion (cm)	Algometry (kg/cm^2^)
The mean difference EG-CG	1.0 ± 0.4 (−1.1, 1.0) p = 1	1.2 ± 1.6 (−2.9, 5.3) p = 0.92	1.1 ± 2,4† (−1.2, 3.5)† p = 0.83
Inside the EG	0.7 ± 0.3 (0.0, 1.5) p = 0.03*	4.8 ± 0.9 (2.5, 7.0) p < 0.001*	1.9 ± 1.3† (1.4, 2.5)† p = 0.67
Inside the CG	0.4 ± 0.3 (−0.4, 1.2) p = 0.57	1.4 ± 1.3 (−2.0, 4.8) p = 0.75	0.6 ± 0.8† (0.2, 1.0)† p = 1

Results are presented as mean ± standard deviation (95% confidence interval).

Legend: EG = experimental group; CG = control group; *p* = level of significance; * = statistically significant (*p* ≤ 0.05); † = data before logarithmic transformation.

**TABLE 3:** Effects at the end of the 4-week FR program – comparison of results after 4 weeks vs. before the first FR.

	Flexion (cm)	Lateral flexion (cm)	Algometry (kg/cm^2^)
The mean difference EG-CG	1.1 ± 0.3 (0.4–1.8) p < 0.001*	5.8 ± 0.9 (3.6, 8.1) p < 0.001*	4.3 ± 2.5† (1.7, 6.8)† p < 0.001*
Inside the EG	1.5 ± 0.3 (0.7–2.3) p < 0.001*	7.3 ± 1.0 (4.8, 9.8) p < 0.001*	4.8 ± 2.8† (3.5, 6.0)† p < 0.001*
Inside the CG	0.0 ± 0.1 (−0.3–0.4) p = 0.99	−0.7 ± 0.4 (−1.8, 0.4) p = 0.38	0.3 ± 1.8† (−0.5, 1.1)† p = 0.11

Results are presented as mean ± standard deviation (95% confidence interval).

Legend: EG = experimental group; CG = control group; *p* = level of significance; * = statistically significant (*p* ≤ 0.05); † = data before logarithmic transformation.

**TABLE 4:** Long-term effects–comparison of results after 1, 3 or 6 months vs. before the first FR.

	Flexion (cm)			Lateral flexion (cm)			Algometry (kg/cm^2^)		
	1 month	3 months	6 months	1 month	3 months	6 months	1 month	3 months	6 months
The mean difference EG-CG	0.4 ± 0.4 (−0.8, 1.6) p = 0.91	1.1 ± 0.3 (0.3, 2.0) p < 0.01*	1.1 ± 0.3 (0.2, 2.0) p < 0.01*	2.8 ± 1.6 (−1.6, 7.1) p = 0.44	4.9 ± 0.8 (2.6, 7.3) p < 0.01*	4.1 ± 0.9 (1.7, 6.5) p < 0.01*	3.4 ± 2.5† (0.8, 5.9)†p = 0.3	3.9 ± 2.8† (1.1, 6.7)†p = 0.2	3.7 ± 2.6† (1.1, 6.3)†p = 0.04*
Inside the EG	1.2 ± 0.3 (0.3, 2.1) p < 0.01*	1.1 ± 0.3 (0.2, 2.0) p < 0.01*	1.1 ± 0.4 (0.1, 2.1) p = 0.02*	5.4 ± 0.9 (3.0, 7.7) p < 0.01*	3.9 ± 0.9 (1.5, 6.4) p < 0.01*	3.8 ± 1.0 (1.0, 6.6) p < 0.01*	5.1 ± 3.5† (3.5, 6.6)†p = 0.03*	5.3 ± 3.8† (3.5, 7.1)†p < 0.01*	4.5 ± 3.8† (2.7, 6.3)†p = 0.02*
Inside the CG	0.4 ± 0.3 (−0.4, 1.3) p = 0.73	−0.4 ± 0.2 (−0.8, 0.1) p = 0.12	−0.3 ± 0.2 (−0.8, 0.1) p = 0.22	1.4 ± 1.3 (−2.3, 5.1) p = 0.88	−2.2 ± 0.4 (−3.4, −1.0) p < 0.01*	−1.5 ± 0.4 (−2.7, −0.3) p < 0.01*	1.5 ± 2,1† (0.6, 2.5)†p = 1	0.9 ± 2.1† (−0.1, 1.9)†p < 0.01*	0.7 ± 1.8† (−0.2, 1.6)†p = 0.07

Results are presented as mean ± standard deviation (95% confidence interval).

Legend: EG = experimental group; CG = control group; *p* = level of significance; * = statistically significant (*p* ≤ 0.05); † = data before logarithmic transformation.

Cohen’s d value is 1.15 for flexion, 1.06 for lateral flexion and 0.98 for algometry, which represents a large effect size.

In the case of algometry measurements, a problem with heteroskedasticity was identified when testing the assumptions of linear mixed models, so a logarithmic transformation of the data was required before further statistical analysis. At the beginning of the study, there were no statistically significant differences between the groups (p = 0.44). In the EG, we noted an increase in pressure pain threshold after the first FR session, but the results only reached statistical significance after the 4-week FR program (p < 0.001), which persisted in all follow-up measurements (p < 0.03). There was no trend towards an increase in the pressure pain threshold at CG, but statistically significantly, better results were recorded at the measurement after 3 months compared to the initial measurements (p < 0.01). The comparison of the results between the groups showed that the pressure pain threshold in the EG increased statistically significantly at the end of the 4-week program (p < 0.001). A statistically significant difference between the groups was also observed 6 months after the end of the FR program (p = 0.04), but did not reach statistical significance in the interim periods (p ≥ 0.2).

## 4 Discussion

The purpose of this study was to determine the short-term and long-term effects of FR using a foam roller on pressure pain threshold and range of motion of the lumbar spine in healthy subjects. The results of our study showed that FR improves mobility and increases the pressure pain threshold. Lumbar spine mobility increased after just one FR, while after 4 weeks of FR, mobility increased significantly. Pressure pain threshold did not change statistically significantly after one FR, while it increased significantly after 4 weeks of FR. The effects were also noticeable at 1-, 3- and 6-months follow-up. The calculated power of the test for all 43 included subjects is 92%. The observed effect size was large (d ≥ 0.98) for flexion, lateral flexion, and algometry measurements, indicating low variability in results. This is the first study to determine the long-term effects of FR on the range of motion and pressure pain threshold of the lower back in healthy subjects.

After just one FR, we found a short-term improvement in the mobility of the lumbar spine with statistically significant difference for flexion (p = 0.03) and lateral flexion (p < 0.001), while the pressure pain threshold measurements did not reach statistical significance. In a similar study, Griefahn et al. (2017) found no improvement in flexion of the lumbar spine after one FR session. Unlike us, foam rolling was performed only on the area of the gluteus maximus and thoracolumbar spine. Most of the existing research investigates the effects of FR on mobility of the lower limbs. Kasahara et al. (2022) demonstrated an improvement in the mobility of the knee joint after one FR (p < 0.01), which was performed at the front of the thigh. In this research, FR was also performed at a speed of 1 stroke/second, but unlike in our research, subjects were instructed to apply as much pressure to the foam roller as they could tolerate. Skinner et al. (2020) conducted a literature review and meta-analysis of the effects of FR and concluded that while FR has short-term effects on improving mobility, long-term effects have not yet been well studied. Hendricks et al. (2020) agree with these findings. There are differences between studies in the use of tools (different types and hardness) and between foam rolling protocols (speed, duration, number of repetitions, intensity of pressure) therefore, it is more difficult to compare the studies with each other. In contrast to the improvement in mobility, we did not find a statistically significant improvement in the pressure pain threshold at the lower back area after the first FR. The results differ from the findings of Cheatham et al. (2017) who reported an increase in pressure pain threshold at the anterior thigh after a single FR. Unlike us, they used a hard and ribbed roller, performed slower movements at a speed of 1 inch/second, and additionally performed active movements in the knee joint during the exercises. Similarly, (Kasahara et al., 2022) found an increase in the pressure pain threshold at the thigh area after one FR, in which the subjects applied to the foam roller the strongest pressure they could still tolerate. Vaughan et al. (2014) found an increase in pressure pain threshold immediately after FR on the iliotibial tract, but the effects disappeared when re-measured after 5 min. Studies differ from ours in targeted body areas, suggesting that perhaps the acute effects of FR differ depending on the musculature targeted. However, there are considerable differences in treatment protocols between studies, so it is difficult to draw conclusions from this. There is a noticeable trend of an acute increase in the pressure pain threshold in studies that used a hard roller or exerted stronger pressure on the foam roller, but the effects soon wear off (Vaughan et al., 2014; Hendricks et al., 2020; Kasahara et al., 2022). Limited research has aimed to determine the duration of effects following a single FR session, yielding conflicting results. Kasahara et al. (2022) reported an improvement in knee flexion persisting for 30 min after a single FR, while Nakamura et al. (2021b) found that dorsiflexion returned to baseline 30 min after FR. Research has also found that an acutely raised pressure pain threshold returns to its baseline value in 5 min (Vaughan et al., 2014) or 10 min after single FR (Kasahara et al., 2022).

At the end of our 4-week FR program, we recorded a statistically significant improvement in mobility and an increase in the pressure pain threshold of the lumbar spine area for the EG compared to the initial measurements (p < 0.001) as well as compared to the CG (p < 0.001). In a similar study, Kiyono et al. (2022) reported a statistically significant (p < 0.05) improvement in dorsiflexion after performing FR on the posterior calf 3 times a week for 5 weeks. Junker and Stöggl. (2015) also demonstrated that using FR 3 times a week for 4 weeks improves the mobility of the hamstring muscles (p < 0.001).

Our results show that there are a long-term effects of 4-week FR program on improvement in mobility and an increase in the pressure pain threshold of the lower back. Effects persisted for up to 6 months after cessation of FR program and were statistically significantly better in the EG compared to the CG (p ≤ 0.04) and within the EG itself compared to the initial measurements (p ≤ 0.03). On average, the subjects achieved the best results right at the end of the 4-week FR program and the results decreased with time, but after 6 months they were still statistically significantly better than in the CG.

The short-term and long-term effects of FR have not yet been well understood. The improvement of mobility seen in our study could be attributed to the improvement in fascial sliding (Griefahn et al., 2017), changes in the water content (Schleip et al., 2012), reduction in muscle stiffness (Stecco et al., 2020), thixotropic nature of the tissue (Schleip, 2003; Behm and Wilke, 2019), piezoelectric stimulation of connective tissue (Schleip, 2003), release of fascial adhesions (Hedley, 2010), tissue adaptation according to the tensegrity model (Jarmey and Myers, 2006), reduction of fascial inflammation (Bednar et al., 1995) and release of myofascial trigger points (Bron and Dommerholt, 2012). Griefahn et al. (2017) investigated the short-term effects of foam rolling on the mobility of the thoracolumbar fascia using ultrasound diagnostics. They found a statistically significant (p < 0.001) improvement in fascial mobility immediately after foam rolling. These results are consistent with Schleip et al. (2012) theory that the mechanical pressure applied by a foam roller causes changes in water content and stimulates hydration of the fascia, which consequently becomes more lubricated and elastic. Mobility could also improve due to a reduction in muscle stiffness. Wilke et al. (2019) used a semi-electronic tissue compliance meter to determine whether FR influences changes in tissue stiffness of the anterior thigh tissue stiffness. A 15%–24% reduction in stiffness was noted after FR, with a greater difference 10 min after FR than immediately afterwards. FR is thought to influence tissue stiffness, but the effects are observed with a delay (Behm and Wilke, 2019). The reduction in muscle stiffness is also influenced by the thixotropic nature of the tissue. During FR, direct pressure is applied to the skin, fascia, muscles and tendons via the foam roller, causing friction. As a result, the temperature of the treated tissue increases and the shear stress increases, leading to a reduction in the viscosity of the intra- and extracellular fluid and consequently less resistance to movement. It is assumed that thixotropic effects can therefore contribute to improved mobility (Schleip, 2003; Behm and Wilke, 2019).

Many of the aforementioned mechanical mechanisms have been criticized on the grounds that the deformation of most tissues would require very strong pressure beyond the limits of normal human capabilities (Chaudhry et al., 2008) and that they alone cannot explain all the effects that occur during FR, especially when it comes to non-localized effects of the FR. Therefore, an increasing number of studies tend to suggest that neurophysiological mechanisms are to the greatest extent responsible for the observed effects of the FR (Beardsley and Škarabot, 2015; Behm and Wilke, 2019; Konrad et al., 2022). They can be divided into two main groups. The first involves the Golgi reflex arc, while the second focuses on the mechanisms of action of other mechanoreceptors, such as Ruffini endings, Pacinian bodies and interstitial muscle receptors, which are often found in fascia (Beardsley and Škarabot, 2015; Stecco et al., 2007). One of the proposed mechanisms that could influence the improvement of mobility is an increased stretch tolerance (Weppler and Magnusson, 2010). Exposure to unpleasant or painful stimuli, such as high-intensity FR, can globally increase tolerance to stretch or pain and thereby increase pressure pain threshold, which could at least to some extent contribute to improved mobility (Behm and Wilke, 2019; Hendricks et al., 2020). However, to increase tolerance, excessive pain is not necessary when performing FR. Grabow et al. (2018) demonstrated that different intensities of pressure on the foam roller did not have a different effect on the range of motion, and that even less painful FR improved mobility. Studies have often shown global or non-local changes after FR. Fauris et al. (2021) performed FR on five different areas of the superficial back myofascial line in a study with 94 subjects. They found that the mobility of the posterior thigh muscles improved statistically significantly in the subjects, regardless on which area of the superficial back line the FR was applied to. Studies have also shown a contralateral improvement after FR in mobility (Killen et al., 2019; Nakamura et al., 2021a) and an increase in pressure pain threshold (Aboodarda et al., 2015; Nakamura et al., 2021a) in the opposite, untreated lower limb. Those contralateral effects suggest the involvement of the central nervous system in modulation of pain. The proposed mechanisms of action relate to the gate theory, diffuse noxious inhibitory control, and the parasympathetic nervous system, which globally influence the inhibition of pain perception, which could also contribute to the improvement of mobility (Behm and Wilke, 2019). The FR program is designed to influence the release of soft tissue structures in the lower back. However, it should be kept in mind that the study was conducted on healthy, asymptomatic subjects. This means that there should be no previous “constriction” or “tightening” of the fascia, as this would otherwise lead to pain, limited mobility or other symptoms (Myers, 2014; Stecco et al., 2006). The results are therefore most likely not due to a relaxation of the myofascia, but to a decrease in the basic muscle tone and an increase in the extensibility of the connective tissue, which led to an increase in mobility, which, however, remained within physiological limits (Beardsley and Škarabot, 2015; Behm and Wilke, 2019).

Although in our study we did not research the exact mechanisms of action of FR, which cause the described effects, and consequently they are not known to us, the above-mentioned processes could be responsible for the observed changes in our case as well.

Despite this study’s novelty, we acknowledge its several limitations. First one is the absence of a blinded investigator and a blinded subject, which could influence our results. In some studies, CG subjects were given a foam roller with instructions to apply minimal pressure to it during exercise, but some effects were also found after such sham sessions (Aboodarda et al., 2015). Another one is that the subjects applied pressure on a foam roller up to 3/10 pain score on a numeric rating scale, which is a subjective rating. The pressure could be better standardized if the test subjects performed the exercises on a force plate, as was done in the study by Yoshimura et al. (2021). The subjects exerted a pressure of 15%–25% of their body weight on the foam roller and monitored it on the computer throughout the FR session. Next limitation is that the subjects performed the FR program alone at home, without supervision, which means we cannot know for sure whether they actually performed the FR to its full extent and correctly as we showed them on the first day. To minimize the mentioned risk, we gave them written instructions with pictures and attached a calendar for monitoring the implementation of the exercises. Another limitation is that at the last measurements, 6 months after the end of the program, we recorded a dropout of 16.3% of the subjects, which could affect the obtained long-term results.

This is the first study to determine the long-term effects of FR on the range of motion and pressure pain threshold of the lower back in healthy subjects. It would be beneficial if future studies compared our FR program to a CG that would perform standard techniques to increase mobility, such as stretching exercises. Future research should also seek to determine the optimal parameters of FR, regarding the use of the tools (foam rollers vs. roller massagers, harder vs. softer tools, ribbed vs. smooth surface, vibrating vs. non-vibrating) and regarding the FR protocols themselves (speed, duration, number of repetitions, intensity of pressure) and also to determine the mechanisms of action of FR, especially after a longer period of exercises. Given our positive results on improving mobility and increasing the pain threshold in healthy subjects, it would be beneficial to repeat the study in subjects with chronic low back pain.

In conclusion, this study showed that FR with a foam roller on the superficial back line improves mobility and increases pressure pain threshold of the lumbar spine in healthy subjects. Positive effects were achieved by performing FR 3 times a week, for 4 weeks, using a soft foam roller and performing rapid strokes (1 stroke/second). Short-term effects after just one FR were noticed for improvement of mobility, while after 4-weeks of FR program there were significant improvements in mobility as well as in pressure pain threshold with long-lasting effects that were seen for up to 6 months. Our results could benefit the athletic population, as a warm-up method or as part of a training program to improve mobility and increase pressure pain threshold, potentially influencing long-term athletic performance and injury prevention. Given our positive results, they could also be useful in the development of new methods of treating low back pain.

## Data Availability

The raw data supporting the conclusions of this article will be made available by the authors, without undue reservation.
