# Supplementary material for: Effects of lower back foam rolling on the pressure pain threshold and the range of motion of the lumbar spine in healthy individuals
Source: Front Physiol. 2024 Sep 25;15:1476342. doi: 10.3389/fphys.2024.1476342 (PMC11461969; doi:10.3389/fphys.2024.1476342)
Supplement: Supplementary file 3 [file DataSheet1.pdf]

Supplementary Material

The SMFR intervention consisted of the following five exercises:

Exercise 1: Plantar fascia area. The test subjects stood with a ball under their foot. They rolled the middle part of the foot five times, the outer part five times and the inner part five times along the rays of the plantar fascia. Then they pressed their heel on the ball ten times (Figure 1). The entire exercise was repeated three times for each foot.

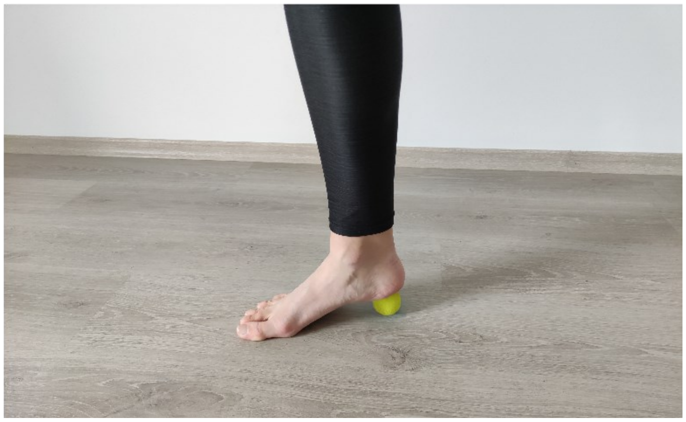

Figure S1. SMFR at the plantar fascia area (author: Julia Fijavž).

Exercise 2: Posterior calf area (Figure 2). The exercise was performed sitting on the floor. The foam roller was placed under the calf of the outstretched leg and the opposite leg was bent. The test subjects lifted their buttocks into the air and rolled the entire calf, from the ankle to the knee, up and down the middle part three times. Then they turned the foot inwards and rolled the calf three times on the inner side. Finally, they turned the foot outwards and rolled the calf three times on the outer side. The exercise was then repeated with the other leg.

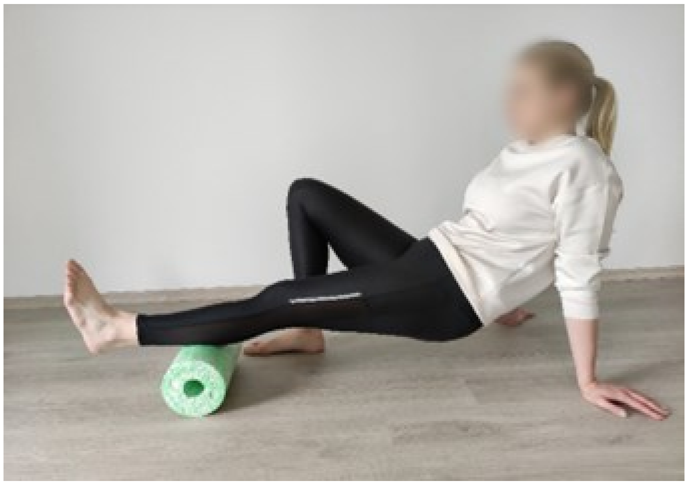

Figure S2. SMFR at the posterior calf area (author: Julia Fijavž).

Exercise 3: Posterior thigh area (Figure 3). The exercise was performed sitting on the floor. The foam roller was placed under the thigh of the outstretched leg, the opposite leg was bent. The subjects lifted their buttocks into the air and rolled the entire thigh, from the knee to the buttocks, up and down the middle part three times. Then they turned the foot inwards and rolled the thigh three times on the inner side. Finally, they turned the foot outwards and rolled the thigh three times on the outer side. The exercise was then repeated with the other leg.

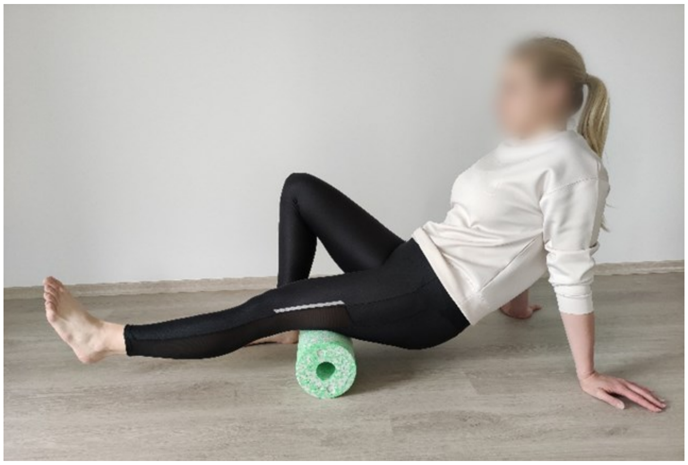

Figure S3. SMFR at posterior thigh area (author: Julia Fijavž).

Exercise 4: Gluteal area (Figure 4). The exercise was performed sitting on a foam roller. The hands were placed on the floor behind the body for support, the left ankle was placed on the bent right knee, the weight was shifted to the left side of the buttocks and the entire area was rolled back and forth eight times from the ischial tuberosity to the iliac crest. The exercise was then repeated on the opposite side.

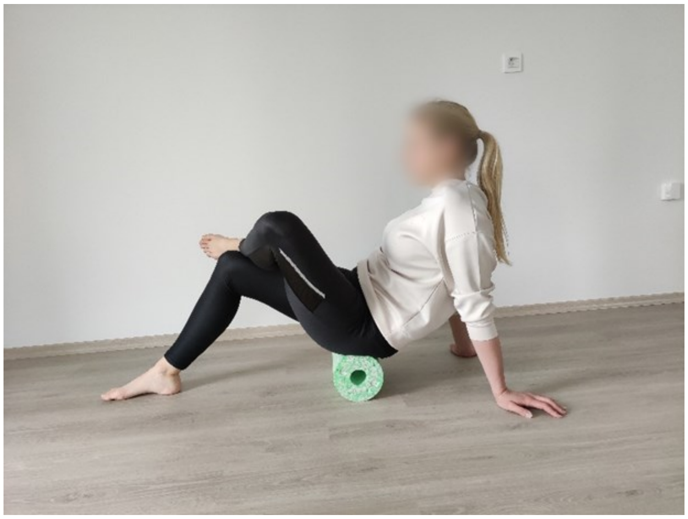

Figure S4. SMFR at gluteal area (author: Julia Fijavž).

Exercise 5: Lower back area (Figure 5). The test subjects stood with their backs to the wall. The foam roller was placed between their lumbar region and the wall. They bent and straightened their legs so that they could roll the entire lower part of the spine from the buttocks to the level of the shoulder blades. The exercise was repeated eight times.

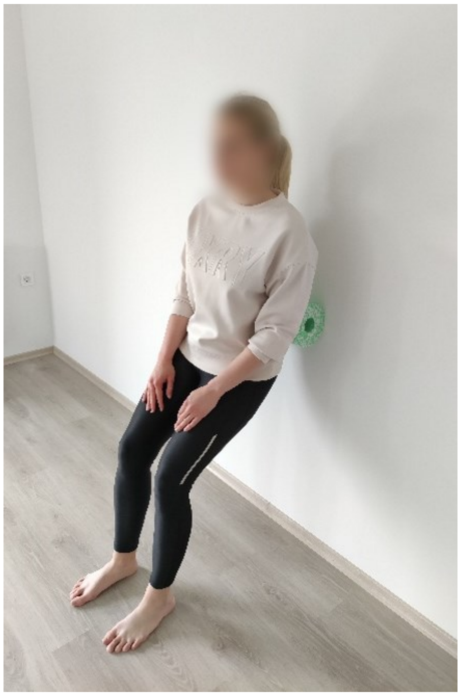

Figure S5. SMFR at the lower back area (author: Julia Fijavž).
